# Elimination of Human Papillomavirus 16-Positive Tumors by a Mucosal rAd5 Therapeutic Vaccination in a Pre-Clinical Murine Study

**DOI:** 10.3390/vaccines12090955

**Published:** 2024-08-23

**Authors:** Molly R. Braun, Anne C. Moore, Jonathan D. Lindbloom, Katherine A. Hodgson, Emery G. Dora, Sean N. Tucker

**Affiliations:** 1 Vaxart Inc., 170 Harbor Way Suite 300, South San Francisco, CA 94080, USA; anne.moore@ucc.ie (A.C.M.); edora@vaxart.com (E.G.D.); stucker@vaxart.com (S.N.T.); 2 School of Biochemistry and Cell Biology, University College Cork, T12 XF62 Cork, Ireland; 3 National Institute of Bioprocessing Research and Training, A94 X099 Dublin, Ireland

**Keywords:** human papillomavirus, therapeutic vaccination, mucosal immunity, CD8+ T cell responses

## Abstract

Therapeutic vaccination can harness the body’s cellular immune system to target and destroy cancerous cells. Several treatment options are available to eliminate pre-cancerous and cancerous lesions caused by human papillomaviruses (HPV), but may not result in a long-term cure. Therapeutic vaccination may offer an effective, durable, and minimally intrusive alternative. We developed mucosally delivered, recombinant, non-replicating human adenovirus type 5 (rAd5)-vectored vaccines that encode HPV16′s oncogenic proteins E6 and E7 alongside a molecular dsRNA adjuvant. The induction of antigen-specific T cells and the therapeutic efficacy of rAd5 were evaluated in a mouse model of HPV tumorigenesis where E6E7-transformed cells, TC-1, were implanted subcutaneously in C57BL/6 mice. After tumor growth, mice were treated intranasally with rAd5 vaccines expressing the wildtype form of E6E7 (rAd5-16/E6E7_Wt_) in combination with an anti-PD-1 antibody or isotype control. Animals treated with rAd5-16/E6E7_Wt_ with and without anti-PD-1 had significant reductions in tumor volume and increased survival compared to controls. Further, animals treated with rAd5-16/E6E7_Wt_ had increased CD4+ and CD8+ tumor-infiltrating lymphocytes (TILs) and produced a cytotoxic tumor microenvironment. In a second study, the immunogenicity of a non-transformative form of E6E7 (rAd5-16/E6E7_Mu_) and a vaccine encoding predicted T cell epitopes of E6E7 (rAd5-16/E6E7_epi_) were evaluated. These vaccines elicited significant reductions in TC-1 tumor volume and increased survival of animals. Antigen-specific CD8+ T effector memory cells were observed in the animals treated with E6E7-encoding rAd5, but not in the rAd5-empty group. The work described here demonstrates that this mucosal vaccination can be used therapeutically to elicit specific cellular immunity and further identifies a clinical candidate with great potential for the treatment and prevention of human cervical cancer.

## 1. Introduction

Most cervical cancers, as well as oropharyngeal and anogenital cancers, are caused by persistent infection with human papillomavirus (HPV) [1]. There are over 200 types of HPV, with HPV16 and HPV18 being responsible for 71% of cervical cancers [2]. Prophylactic vaccines against the viral L1 capsid protein are currently in use and are highly protective against the most common and high-risk HPVs (hrHPVs), including HPV16. However, these vaccines are only effective if administered prior to infection and have no therapeutic effects [3]. Most infections are spread through sexual contact and are cleared by the body without intervention, with persistent infection occurring in approximately 10% of patients [4]. The development of cervical intraepithelial neoplasia (CIN), abnormal changes in the cells lining the cervix, is caused by these persistent infections. Severity is graded by a scale from CIN1 to CIN3, with CIN3 being the most severe form. When left untreated, these abnormal cell pathologies can lead to cervical cancer [5]. Currently, the standard of care to treat CIN2-3 involves invasive surgical methods such as ablation or excision of a large section of the cervix [6]. While these methods have worked well when employed properly and in a timely manner, treatment may not result in durable protection. Immunotherapy treatments via a therapeutic vaccine can specifically target precancerous and malignant cells that express HPV oncoproteins, generating a more targeted approach to treatment. Further, by training the immune system to target HPV+ cells, therapeutic vaccination may provide more durable protection from re-occurrence than traditional methods of treating CIN2-3 [7]. Excitingly, there have been many recent advances in the field of immunotherapy, particularly in therapeutic vaccination, which aims to provide non-invasive, more durable treatments to prevent progression to cervical cancer. Previous work using therapeutic vaccination to treat cervical cancer has shown efficacy correlated with a robust HPV-specific T cell response [8]. Therefore, a successfully implemented therapeutic vaccine must be able to stimulate these immune responses.

HPV is a double-stranded circular DNA virus. During infection, viral proteins E6 and E7 modify the cell cycle to promote viral genome amplification using the host cell’s machinery. In some cases, the viral genome may integrate with the host genome, allowing the continual presence of these proteins [9,10]. Unchecked expression of E6 and E7 causes unregulated cell entry into S-phase, leading to oncogenesis. As E6 and E7 are the causative agents of pre-cancerous and cancerous pathologies, stimulation of cytotoxic cellular immunity specific to these proteins has often been the objective of therapeutic vaccination [11]. Many studies have demonstrated that targeting these two antigens can lead to tumor and/or lesion regression and viral clearance [12,13,14,15,16,17,18,19,20,21]. We have developed a mucosal vaccine platform known as Vector-Adjuvant-Antigen Standardized Technology (VAAST^®^), which utilizes non-replicating recombinant human adenovirus type 5 (rAd5) to express an antigen of interest as well as a molecular dsRNA adjuvant within the same target cell [22,23,24,25,26]. The dsRNA forms a short hairpin RNA structure that is expressed independently from the same rAd5 vector as the antigen of interest. This hairpin structure can stimulate cellular innate immune sensors when expressed within a cell, directing an immune response to the very cell where the antigen is expressed, and increase overall immunogenicity. When used in clinical trials, these vaccines are formulated into enterically coated tablets that can be administered orally to humans to stimulate mucosal and systemic immune responses [22,23,24,25,26,27]. After delivery to the ileum [25], rAd5 is believed to be taken up by epithelial and resident immune cells where the transgene is expressed in the context of MHC I or cross-presented on dendritic cells via MHC I and MHC II. Effector T cells recognize the presented vaccine antigens and elicit cell-mediated immunity in the mucosa [28]. Despite delivery to the ileum, evidence of mucosal activation and transit throughout the body has been observed in humans using this platform. In a phase II influenza challenge study, oral rAd5 encoding the influenza haemagglutinin gene was administered prior to pandemic H1 influenza challenge. The vaccine elicited HA-specific antibodies, mucosal homing B cells, and protected subjects from intranasal challenge with influenza [23]. Additionally, phase I and phase II clinical trials have shown antigen-specific antibodies in saliva and nasal secretions [29,30] as well as upregulation of the mucosal homing marker α4β7+ on B and T cells [22,23]. Therefore, although this oral tablet vaccine is administered in humans via the intestinal mucosa, there is direct evidence of mucosal cross-talk beyond the intestinal site of delivery.

In recent years, the use of checkpoint inhibitors (CPIs), antibodies that block inhibitory signals between antigen-presenting cells and cytotoxic T cells, have been widely used to treat a variety of cancers including lung cancer, bladder cancer, and head and neck cancers [31], in addition to malignant cervical cancer [32]. One main target of these CPI therapies is programmed cell death protein 1 (PD-1), which is expressed on CTLs and, when activated via engagement of PD-L1, dampens the cytotoxic capacity of T cells. This pathway is often exploited by tumor cells to suppress the local immune response [33]. Expression of PD-1 and its receptor PD-L1 has been found in cells from patients with cervical cancer [34]. Previous studies have demonstrated that there are advantages in combining CPIs with therapeutic vaccination [19,35,36,37]. Therefore, the addition of antibodies targeting PD-1 to immunotherapies may improve disease outcomes.

In this study, we examined the antitumor effects of mucosal therapeutic vaccination with rAd5 encoding the HPV16 genes E6 and E7. rAd5 was tested with anti-PD-1 antibodies to examine if the combination of vaccination with a CPI could enhance the therapeutic efficacy of treatment. HPV16 does not infect animals nor lead to the mucosal tumorigenesis seen in humans, limiting methods to study therapeutic vaccination pre-clinically [38]. In a proof-of-concept experiment, we employed a commonly available HPV tumorigenesis model that uses the TC-1 cell line, murine C57BL/6 lung cells generated by transduction with HPV16 E6, E7, and the variant H-ras activated by the G12V mutation [39,40]. These cells can be cultured in vitro and implanted subcutaneously into C57BL/6 mice, providing a surrogate model to test therapeutic vaccinations [19,39]. Intranasal vaccination was used as a mucosal proxy for oral delivery as oral gavage methods deliver the vaccine to the stomach, rather than the intestines, leaving rAd5 susceptible to the stomach’s low-pH environment [41]. However, previous studies with rAd5 vaccines show that immunological readouts in mice aligned with positive immunogenicity readouts in human clinical trials [27,42].

We found that the HPV16-specific rAd5, when administered to TC-1 tumor-bearing mice, led to a significant reduction in tumor volume and increased survival. This was true when the vaccinating antigen was the wildtype form of E6 and E7, encoded selected mutations, or was co-administered with anti-PD-1. Tumor-infiltrating lymphocytes (TILs) were increased in E6E7-specific vaccination and generated a ratio of Treg/CD8+ T cells that has been previously associated with improved clinical outcomes [43]. Relative percentages of CD4+ and CD8+ T cells remained consistent between the groups treated with E6/E7-containing rAd5 compared to controls; however, there was a significant increase in E7-specific CD8+ T cells in these groups, which was largely made up by T effector memory cells. Our results indicate that these mucosal E6/E7-expressing rAd5 vectors are efficacious and immunogenic in a mouse model of HPV-derived tumorigenesis and may represent an effective, non-invasive, and potent therapeutic for the treatment of HPV-related cancers.

## 2. Materials and Methods

### 2.1. rAd5 Generation

The transgene expressed by the rAd5-16/E6E7_Wt_ vaccine was generated based on the published sequence of HPV16 E6 (GenBank Accession Number ANY26540.1) and E7 (GenBank Accession Number AIQ82815.1). A tPA signal sequence is upstream of the E6E7 gene and a furin cleavage site separates E6 and E7. For rAd5-16/E6E7_Mu_, E6 mutations L57G, E154A, T156A, Q157A, and L158A and E7 mutations H2P, C24G, E46A, and L67R were introduced. rAd5-16/E6E7_Mu.1_ shares the same mutations are rAd5-16/E6E7_Mu_, except for E7 L67R. For rAd5-16/E6E7_epi_, MHC class I binding predictions were made in May 2019 using the IEDB analysis resource ANN tool [44,45]. Amino acids 18–26, 42–56, and 126–143 of E6 and 4–23, 62–77, and 81–90 of E7 were included without spacers, furin cleavage sites, or tPA sequences. The transgene sequences described above were inserted into the E1 region of a recombinant plasmid containing the rAd5 genome lacking the E1 and E3 genes. A third rAd5 construct was used that did not contain a transgene sequence (rAd5-empty). A sequence encoding the molecular dsRNA adjuvant was included downstream of the transgene region in all constructs. Both E6/E7 and the dsRNA are under the control of CMV promoters. The rAd5 vaccines were generated and propagated as previously described [46].

### 2.2. ELISpot

C57BL/6 mice (*n* = 2–8/group, female, 6–8 weeks old, Jackson Labs, Bar Harbor, ME, USA) or J:DO mice (*n* = 3–8/group, female, 6–8 weeks old, Jackson Labs), were vaccinated three times one week apart with 1 × 10^8^ infectious units (IUs)/animal. On day 21, one week after final vaccination, 5 × 10^5^ splenocytes were cultured in duplicate wells with a pool of overlapping peptides spanning the coding sequence of E6 or of E7. These peptides, which were synthesized as 15-mers overlapping by 11 amino acids (JPT GmbH, Berlin, Germany), were reconstituted in DMSO and subsequently diluted in RPMI-based cell culture medium at 0.2 µg/well. After overnight incubation, the numbers of spot forming units (SFUs) of IFNγ per million splenocytes was determined. Spots were counted using an AID ELISPOT reader or by Zellnet Consulting (Fort Lee, NJ, USA). Animal work was performed at Vaxart, Inc. (South San Francisco, CA, USA). The animal procedures used in the current study were submitted to the Institutional Animal Care and Use Committee (IACUC).

### 2.3. TC-1 Challenge Model

TC-1 cells (ATCC, Manassas, VA, USA) were grown as a monolayer at 37 °C with 5% CO_2_ in RPMI 1640 supplemented with 2 mM L-glutamine (31870-025, Thermofisher, Waltham, MA, USA), 1 mM sodium pyruvate (11360-039, Thermofisher), 0.1 mM non-essential amino acids (11140-035, Thermofisher), 50 µM β-mercaptoethanol (31350-010, Thermofisher), 1% penicillin/streptomycin (15140-122, Thermofisher), and 10% fetal bovine serum (P30-3306, Pan Biotech, Aidenbach, Germany). For tumor implantation, cells were detached from the plate with trypsin-0.05% EDTA (25300054, Thermofisher) and 1 × 10^6^ TC-1 cells in 200 µL of RPMI 1640 without phenol red were injected into the right flank of 50 C57BL/6JRj mice (female, 7 weeks old, Janiver Labs, Le Genest-Saint-Isle, France). Animals were randomized into groups of 10 by mean tumor volume when tumors reached 20–60 mm^3^ or 100–200 mm^3^ for the small and large tumor models, respectively (Vivo Manager software 1.11E-02, Biosystemes, Couternon, France). All work described was performed by Oncodesign Services (Dijon, France). The animal procedures used in the current study were submitted to the Institutional Animal Care and Use Committee of Oncodesign (Oncomet), approved by French authorities (CNREEA agreement No. 91 (Oncodesign)).

### 2.4. Therapeutic Immunization and Immunotherapy

Therapeutic vaccinations were given by intranasal administration of rAd5 (1 × 10^8^ infectious units (IUs)/animal) on the day of randomization and repeated twice more, seven days apart. The anti-murine PD-1 monoclonal antibody (RMP1-14, BioXcell, Lebanon, NH, USA) or rat IgG2a as an isotype control (2A3, BioXcell) was administered twice weekly during vaccination by intraperitoneal injection at a dose of 10 mg/kg. In experiments with rAd5-16/E6E7_Wt_, animals were treated as follows: (i) PBS; (ii) rAd5-empty + iso; (iii) rAd5-empty + anti-PD-1; (iv) rAd5-16/E6E7_Wt_ + iso; or (v) rAd5-16/E6E7_Wt_ + anti-PD-1. For experiments with modified E6E7, animals were treated as follows: (i) PBS; (ii) rAd5-empty; (iii) rAd5-16/E6E7_Mu_; (iv) rAd5-16/E6E7_epi_. Animals were monitored for clinical signs (viability and behavior) every day. Body weights were measured twice a week. The length and width of the tumor were measured twice a week with calipers and the volume of the tumor was estimated by the following formula: tumor volume = (width2 × length)/2 [47]. Animals were euthanized if the tumor exceeded 10% of the normal body weight or reached 1500 mm^3^. All work described was performed by Oncodesign Services (Dijon, France).

### 2.5. Analysis of Tumor Infiltrating Cells (TILs) by Flow Cytometry

TC-1 tumors were established in the flank as described above and allowed to grow to a mean volume of 111.5 mm^3^ on day 13 post-tumor induction. Five mice per group were randomized into each group and were treated on days 13 and 20 with one of the following regimes: (i) rAd-empty + iso; (ii) rAd-16/E6E7_Wt_ + iso; or (iii) rAd-16/E6E7_Wt_ + anti-PD-1. All doses and routes were the same as previously described. Animals were monitored daily and tumors volumes were determined twice a week. On day 24, all animals were euthanized and tumors were removed, weighed, mechanically disrupted with a scalpel, and then crushed with a 1 mL syringe plunger on a 70 µm sieve. One million cells were resuspended in staining buffer (PBS, 0.2% BSA, 0.02% NaN3). Cells were stained for T cell subsets with FoxP3 PE, CD8a PerCP, CD3 V450, CD4 VioGreen, and CD45 APC-Cy7. The stained cells were analyzed with a flow cytometer (LSR II, BD Biosciences, Durham, NC, USA). Flow cytometry data were acquired until either 50,000 CD45+ events were recorded for each sample or a maximum duration of 2 min elapsed. All work described was performed by Oncodesign Services (Dijon, France).

### 2.6. Flow Cytometry on Blood Cells from Small Tumor Model Animals

Blood was collected by jugular vein puncture of TC-1 mice on days 14 and 21, one day after vaccine treatments. Prior to staining, red blood cells (RBCs) were lysed with Versalyse lysing buffer (A09777, Beckman coulter, Brea, CA, USA) for 10–15 min. Cells were stained with the viability dye Viakrom 808 (Beckman Coulter), followed by staining with Dextramer E749-57 APC (Immudex, Copenhagen, Denmark, Allele H-2Db). Cells were then stained with CD45 BV605 (103155, Biolegend, San Diego, CA, USA), CD3 PE (130-120-160, Miltenyi, Bergisch Gladbach, Germany), CD4 PE-Vio770 (130-123-894, Miltenyi), CD8 BV785 (100750, Biolegend), CD62L (BV421 104436, Biolegend), and CD44 VioBright FITC (130-120-213, Miltenyi). Cells were fixed with Cytofix buffer (554655, BD Biosciences) and then run on a flow cytometer (Cytoflex LX, Beckman Coulter). All work described was performed by Oncodesign Services (Dijon, France).

### 2.7. Statistics

Mean and standard error of the mean (SEM) are shown for all data points except when *n* ≤ 3, in which only mean and individual datapoints are shown. For ELISpot analysis and analysis of blood cells at various timepoints, an ordinary two-way ANOVA with Tukey’s multiple comparison test was used. For TIL analysis, a Mann–Whitney *t*-test was used. Statistics were performed using GraphPad Prism (Version 10.0.3).

## 3. Results

### 3.1. Immunogenicity of Wildtype and Modified E6E7 Therapeutic Vaccines for HPV16

**Figure 1:**
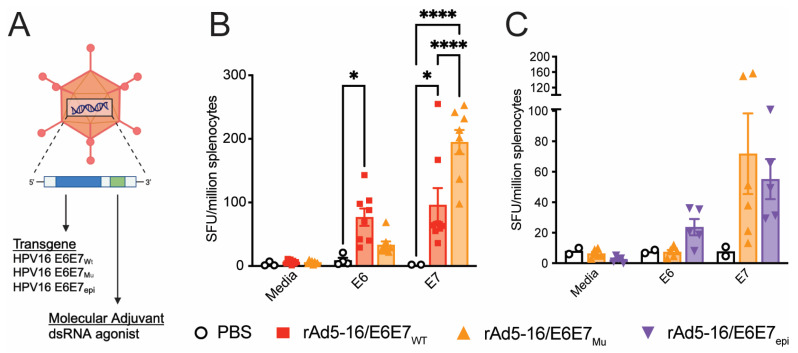
rAd5 vaccines from VAAST platform induce antigen-specific T cells. (**A**) Illustration of the rAd5 vector used during vaccination. The transgene region (blue) represents the antigen included in the construct upstream of the molecular dsRNA adjuvant (green). (**B**,**C**) Antigen-specific T cells expressing IFNγ in C57BL/6 mice intranasally vaccinated with rAd after stimulation with media, E6, or E7 peptide pools. Spot forming units (SFUs) per million spleen cells are shown. Mean and SEM, two-way ANOVA. *n* = 2–8 mice/group. Error bars not shown when *n* ≤ 3. *p* = 0.01 to 0.05 (*), *p* < 0.0001 (****).

The E6 and E7 proteins of HPV16, when unchecked, may cause dysregulation of the cell cycle and tumorigenesis. Several studies have previously mutated HPV16 E6 and/or E7 proteins to reduce the risk associated with the oncogenic potential of these proteins [20,21,48]. Eliminating the oncogenic potential of E6 and E7 in a vaccine could further increase safety and therapeutic acceptance. Therefore, in addition to testing the immunogenicity of wildtype (Wt) E6 and E7 as therapeutic antigens (rAd5-16/E6E7_Wt_), an rAd5 vaccine was generated with mutations that disrupt E6’s ability to bind p53 and PTPN13 and E7’s ability to bind Rb and mi2β (rAd5-16/E6E7_Mu_) (Figure 1A) [21]. In a second approach, a construct was generated to express the minimal epitopes required for immunogenicity and efficacy (rAd5-16/E6E7_epi_), utilizing the Immune Epitope Database & Tools, a resource that uses MHC class I binding, peptide processing, and immunogenicity predictions to identify consequential epitopes [49]. As a comparator, a vaccine was tested which only contains the molecular dsRNA adjuvant (rAd5-empty) (Figure 1A).

Immunogenicity of rAd5 vectors encoding either the wildtype or mutated HPV16 E6E7 were first examined by ELISpot assay to measure specific T cell responses; C57BL/6 mice were intranasally vaccinated on day 0 and day 28. T cells from splenocytes were assessed for antigen-specific IFNγ production one week after the final vaccination. A non-significant decrease in E6 immunogenicity and a significant increase in E7 immunogenicity was observed when comparing rAd5-16/E6E7_Mu_ with rAd5-16/E6E7_Wt_ (Figure 1B). Additionally, no significant differences in T cell responses to E6 or E7 were detected when the mutated E6E7 (rAd5-16/E6E7_Mu_) and minimal epitope vaccine (rAd5-16/E6E7_epi_) were compared in C57BL/6 mice (Figure 1C). E7 has a strong H-2Db-restricted CD8+ T cell epitope (RAHYNIVTF, known as R9F) in C57BL/6 mice that can dominate the immune response. Therefore, we wanted to test if the rAd5 vaccines were immunogenic in outbred mice with more heterologous MHC class I variation. Thus, the outbred mouse strain J:DO was used to provide a better sense of the T cell response with a diverse background of MHC alleles. Mice were vaccinated with rAd5-16/E6E7_Wt_ and a vaccine nearly identical to rAd5-16/E6E7_Mu_ (which differs by one amino acid in E7 L67R, termed rAd5-16/E6E7_Mu.1_) and IFNγ-producing T cells were assessed by ELISpot. Similar trends were observed in J:DO mice as in C57BL/6 mice with no significant differences between E6 responses and significant increases in E7 responses (Figure S1).

### 3.2. Mucosal Application of Antigen-Specific rAd5 Reduces Tumor Size

**Figure 2:**
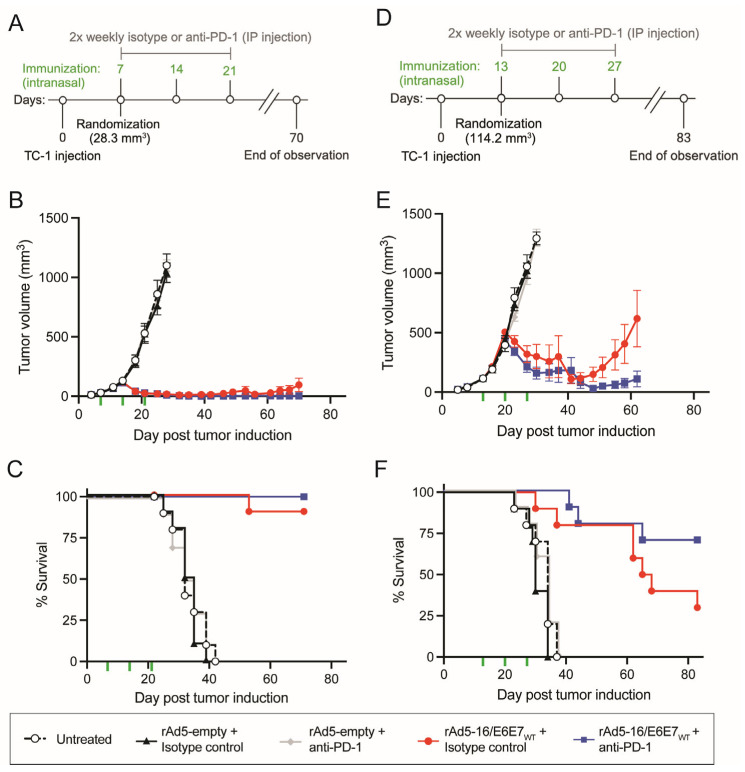
Intranasal administration of rAd5 vaccine expressing wildtype E6-E7 can eliminate and prevent tumor growth in mice. (**A**) Overview of small tumor model timeline. Vaccinations started on day 7 once tumors reached an average of 28.3 mm^3^. (**B**) Tumor volumes were measured in treatment groups through to the study end. Curves are shown for data points where at least 80% of animals were present. Vaccination is indicated by green hash-marks. (**C**) Survival curve of small tumor model indicating time until ethical criteria for euthanasia were met. (**D**) Overview of large tumor model timeline. Vaccinations started on day 13 once tumors reached an average of 114.2 mm^3^. (**E**) Tumor volume in treatment groups through to the study end. Curves are shown for data points where at least 80% of animals were present. Vaccination is indicated by green hash-marks. (**F**) Survival curve of large tumor model indicating time until ethical criteria for euthanasia were met. Mean and SEM. *n* = 10 mice/group.

To test the efficacy of rAd5-16/E6E7_Wt_, we utilized the TC-1 solid tumor growth model. HPV-E6/E7-expressing TC-1 cells were injected subcutaneously into the hind flank of C57BL/6 mice on day 0 and allowed to grow for several days before mice were treated with an intranasal administration of the rAd5-16/E6E7_Wt_ vaccine, rAd5-empty vaccine, or PBS (untreated). In addition to vaccination, mice were administered the CPI monoclonal antibody against PD-1 (anti-PD-1) or an isotype control (Figure 2A,D). These combinations were tested in a small tumor model study, where mice were immunized on day 7 after the tumors had reached a mean volume of 28.3 mm^3^, and then again on days 14 and 21 (Figure 2A). Administration of rAd5-16/E6E7_Wt_ with isotype administration reduced tumor growth and induced tumor shrinkage in all animals up to day 39, at which point 2/10 tumors re-grew and one animal reached the humane endpoint before the study end (Figure 2B,C and Figure S2A). The use of the anti-PD-1 with rAd5-16/E6E7_Wt_ vaccine trended slightly better for survival (10/10 survived), but this result was not significantly different (Figure 2B,C and Figure S2A). Animals treated with rAd5-empty with or without anti-PD-1 or treated with PBS did not control tumor growth and met the ethical criteria for euthanasia before the study end (Figure 2B,C and Figure S2A). Overall, mice immunized with rAd5-16/E6E7_Wt_ overwhelmingly survived the tumor challenge, compared to 0% in the control groups.

To investigate if rAd5 vaccines could have therapeutic efficacy later in tumor progression, TC-1 tumor cells were injected as before but randomization and treatment occurred when tumors reached a mean volume of 114.2 mm^3^ on day 13 (large tumor model). Animals were vaccinated as above, followed by subsequent vaccinations on days 20 and 27 with twice-weekly injections of anti-PD-1 or the isotype control (Figure 2D). The group administered rAd5-16/E6E7_Wt_ with anti-PD-1 was able to control tumor growth, up to day 40, at which point 2/10 mice reached a humane endpoint (Figure 2E,F and Figure S2B). Within this group, 70% (7/10) of animals survived to the end of the experiment on day 83. rAd5-16/E6E7_Wt_ with the isotype control was also able to substantially control tumor growth effects and improve survival with 80% of mice surviving (8/10) to day 60 (33 days after the last immunization), before four additional animals reached a humane endpoint by day 80, resulting in a day 83 survival rate of 30% (Figure 2E,F and Figure S2B). Mice left untreated or treated with rAd5-empty with anti-PD-1 or the isotype control were not able to control tumor growth effects, with all mice in these groups reaching a humane endpoint before day 40 (Figure 2E,F and Figure S2B).

### 3.3. Immunization with Ad-HPV-16 Generates a Cytotoxic Tumor Microenvironment

**Figure 3:**
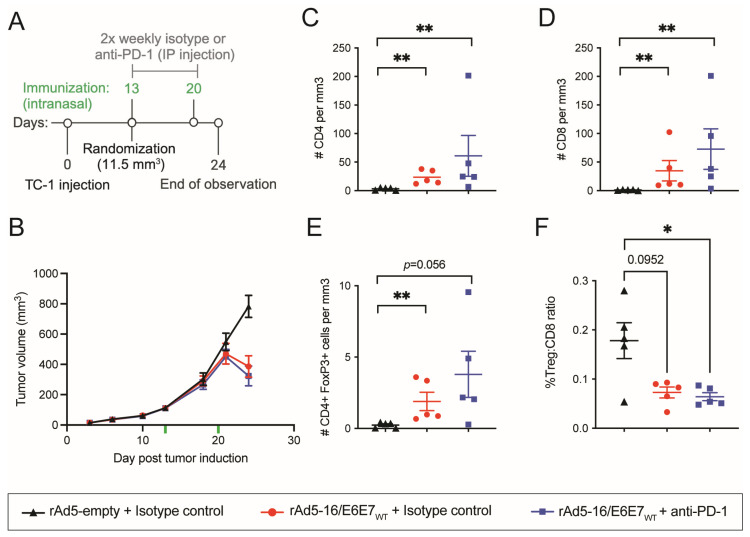
E6E7-specific rAd5 vaccines induce tumor infiltrating lymphocytes. (**A**) Overview of large tumor model timeline for TIL analysis. (**B**) Mean tumor volume per group until tumor collection. Treatment began when mean tumor volumes reached 111.5 mm^3^ on day 13. Vaccination is indicated by green hash marks. (**C**) number of CD4 T cells per mm^3^ of tumor. (**D**) number of CD8 T cells per mm^3^ of tumor. (**E**) number of Treg cells per mm^3^ of tumor. (**F**) Ratio of Treg T cells to CD8 T cells. *n =* 5 mice/group. Mean and SEM. B-D Mann–Whitney *t*-test. *n* = 5 mice/group. *p* = 0.01 to 0.05 (*), *p* = 0.001 to 0.01 (**).

Tumors can elicit Treg cells to dampen the local immune response and promote further tumor growth [50]. It was previously observed that the TILs of individuals who had a higher ratio of Tregs to CD8+ T cells had unfavorable clinical outcomes [43]. To understand how E6E7-specific rAd5 vaccines were able to control tumor growth, TILs were analyzed by flow cytometry using the large tumor model. TC-1 tumor cells were injected and allowed to grow to a mean volume of 111.5 mm^3^ before intranasal immunization with rAd5-16/E6E7_Wt_ with or without anti-PD-1 and compared to immunization with rAd5-empty on day 13 post TC-1 injection (Figure 3A). Mice were vaccinated again seven days later and tumors were harvested on day 24, when initial tumor regression was observed but tumors were still of sufficient mass to permit cell isolation and analysis (Figure 3A,B). Lymphocytes were normalized per unit volume of tumor isolated to allow comparisons across groups. rAd5-16/E6E7_Wt_ with or without the anti-PD-1 checkpoint inhibitor induced a significant increase in the number of CD4+ and CD8+ T cells in the tumor mass along with an increase in Treg cells (defined as CD45 + CD3 + CD4 + FoxP3+) (Figure 3C–E). Although there was in increase in Treg cells in addition to CD8+ T cells in vaccinated animals, the increase in the latter was much greater. Therefore, a lower Treg/CD8+ T cell ratio was observed in rAd5-16/E6E7_Wt_, in the presence or absence of anti-PD-1, compared to rAd5-empty and the isotype antibody (Figure 3F). This demonstrates that immunization with rAd5-16/E6E7_Wt_ alone or in combination with anti-PD1 modulates the inter-tumoral immune response towards an overall cytotoxic tumor microenvironment.

### 3.4. Vaccination with rAd5 Vectors Expressing Non-Oncogenic E6E7 Led to Tumor Reduction

**Figure 4:**
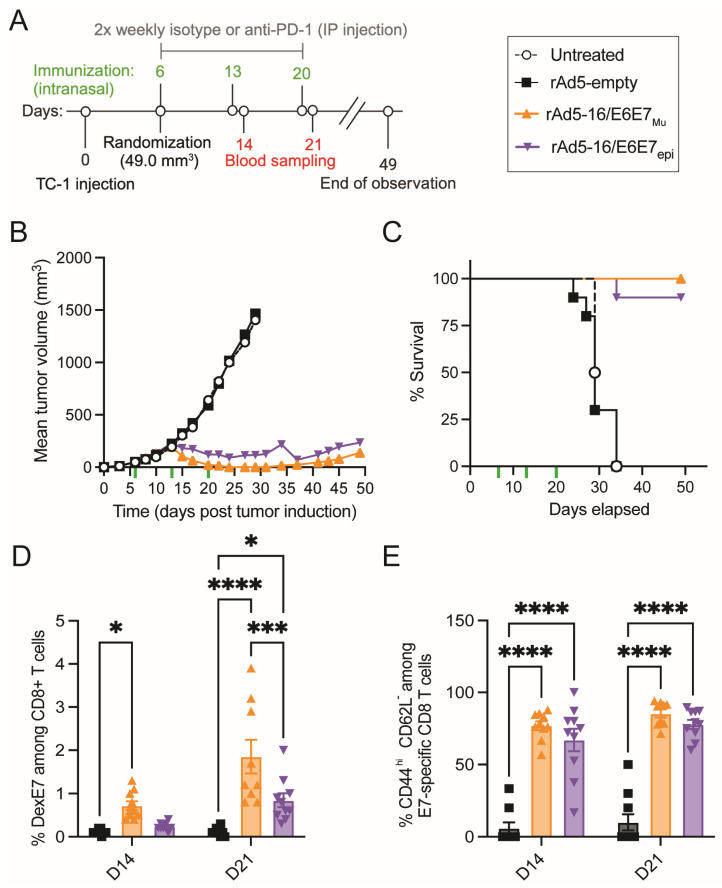
E6-E7-specific rAd5 vaccines with oncogenic mutations prevent tumor growth in mice bearing subcutaneous TC-1 tumors and generate antigen-specific T_EM_ cells. (**A**) Overview of small tumor model timeline. Vaccinations, indicated by green hash marks, started on day 6 once tumors reached an average of 49.0 mm^3^. (**B**) Tumor volume in treatment groups through to the study end. Curves are shown for data points where at least 80% of animals were present. (**C**) Survival curve of small tumor model indicating time until ethical criteria for euthanasia were met. (**D**) Percentage of CD8+ T cells specific to HPV16 E7. (**E**) Percentage of E7-specfic T cells among E7-specific cells. *n* = 10 mice/group, mean and SEM, two-way ANOVA. *p* = 0.01 to 0.05 (*), *p =* 0.001 to 0.0001 (***), *p* < 0.0001 (****).

Although rAd5-16/E6E7_Mu_ and rAd5-16/E6E7_epi_ were immunogenic in ELISpot assays (Figure 1C and Figure S1), we sought to understand if mutating the key oncogenic sites of E6 and E7 in the vaccine affected the ability of the vaccine to elicit an efficacious immune response. Thus, the small tumor model utilized in Figure 2A,B was employed with these constructs. On study day 0, TC-1 tumors were induced by subcutaneous injection of 1 × 10^6^ TC-1 cells into the right flank of C57BL/6 mice. Animals were randomized into treatment groups when tumors reached 49.0 mm^3^ on day 6. Groups were then treated with PBS (untreated), rAd5-empty, rAd5-16/E6E7_Mu_, or rAd5-16/E6E7_epi_ by intranasal application three times, seven days apart (Figure 4A). Blood samples were taken on days 14 and 21 for flow cytometry analysis. After randomization and initial treatment on day 6, tumors continued to grow until day 13, at which point there was no significant difference in tumor size between treatment groups (Figure 4B,C and Figure S2D). Animals received additional treatments on day 13 and day 20. After day 13, tumors in the untreated group and the group treated with rAd5-empty continued to increase in volume until humane endpoints were reached, whereas tumor volume in the groups treated with rAd5-16/E6E7_Mu_ or rAd5-16/E6E7_epi_ began to decrease in size through to the end of the study at day 49 (Figure 4B and Figure S2D). The effect of rAd5-16/E6E7_Mu_ was particularly pronounced as tumor volume completely regressed in all animals with lasting regression in 5/10 animals (Figure S2D).

### 3.5. Mucosal Application of Antigen-Specific rAd5 Generates Antigen-Specific T_EM_ Cells in a Small Tumor Model

As oncogenesis is driven by E6 and E7, which are intracellular targets, cellular immunity after rAd5-16/E6E7_Mu_ and rAd5-16/E6E7_epi_ treatment was characterized by flow cytometry one day after the second and third treatment (days 14 and 21) to look for changes in T cell distributions as well as the generation of antigen-specific T cells (Figure 4A,D,E). Blood was sampled from animals from the group treated with rAd5, but not the untreated group. The percentage of CD3+ cells within the CD45+ population was consistent between groups, except for a statistically significant increase in overall percentage of T cells in the group treated with rAd5-16/E6E7_Mu_ compared to rAd5-empty at day 14. This difference was not present at day 21, one day after the second treatment (Figure S3A). Among CD3+ T cells, there were no differences in overall distribution of either CD8+ T cells or CD4+ T cells at either sampling day between the various groups (Figure S3B,C).

To further characterize the cellular immune response, we next examined the cellular markers CD44 and CD62L in the T cell population to distinguish between naïve, central memory (T_CM_), and effector memory (T_EM_) T cells. There were no significant differences or consistent trends in the distribution between CD8+ T cell subsets (Figure S3D–F). As T_EM_ cells are the main drivers of cellular cytotoxicity towards cancerous cells, we sought to understand if E7-specific T cells were being produced by rAd5 treatments using a dextramer with the known R9F (E749-57) H-2 Db allele (DexE7) bound to MHC class I. On day 14, after two treatments, the rAd5-16/E6E7_Mu_ group had a significant increase in E7-specific T cells compared to the rAd5-empty group. At day 21, after three treatments, both the rAd5-16/E6E7_Mu_ group and the rAd5-16/E6E7_epi_ group had significantly increased percentages of DexE7-specific CD8+ T cells with rAd5-16/E6E7_Mu_ having a significantly higher response to both rAd5-empty and rAd5-16/E6E7_epi_ (Figure 4D). Of the DexE7+ CD8+ T cells, most cells were T_EM_ cells, representing a critical cell population for cell-mediated control of HPV tumorigenesis (Figure 4E).

## 4. Discussion

Therapeutic vaccination, designed to stimulate cell-mediated immunity, may provide revolutionary new options for the treatment of many cancers by stimulating life-long memory anti-tumor responses. HPV-specific therapeutic vaccination may also provide a less invasive, more easily administered, and more widely available treatment for CIN2/3. Here, we demonstrate that mucosally administered adenovirus-based vaccines expressing HPV16 antigens are highly efficacious at killing HPV16-immortalized epithelial cells in a mouse model. These vaccines induced high levels of antigen-specific T cells in inbred and outbred mice and induced a favorable ratio of cytotoxic to regulatory T cells in tumors, overall leading to efficacy and animal survival in the TC-1 tumorigenesis murine model.

The first objective of this study was to determine the immunogenicity of candidate HPV16 E6 and E7 antigens expressed by the adenovirus type 5 vector. We show that E6E7-specific rAd5 vaccination leads to the generation of specific T cell responses in C57BL/6 and J:DO mice. Mutations in the wildtype antigen led to some changes in the measured T cell responses in C57BL/6 mice. Specifically, when comparing rAd5-16/E6E7_Wt_ and rAd5-16/E6E7_Mu_, the latter yielded a decreased SPU count after E6 stimulation, whereas an increase in SPU was observed after E7 stimulation in splenocytes from vaccinated C57BL/6 mice. It is possible that the specific mutations in rAd5-16/E6E7_Mu_ change the peptide processing for MHC class I presentation. For example, an E6 intermediate affinity epitope, identified by the IEDB analysis resource ANN tool, is present between amino acid residues 52–60 in wildtype HPV16 E6 (FAFRDLCIV). The L57G substitution of rAd5-16/E6E7_Mu_ may disrupt this H-2Db epitope, leading to a reduced T cell response. This was not, however, observed when tested in J:DO mice. It could additionally be speculated that the E7 E46A mutation in rAd5-16/E6E7_Mu_, which is three positions upstream of the dominant H-2Db RAHYNIVTF epitope, further enhances processing and presentation. Future studies are required to determine the relative contribution of predicted H-2Db and H-2Kb epitopes to the overall T cell responses to these antigens.

Importantly, we show that rAd-16E6E7 vaccines drive tumor regression at both early and late stages of tumor growth and generate a cytotoxic tumor microenvironment. The addition of the anti-PD-1 antibody increased the durability of the vaccine-induced anti-tumor response by at least 20 days in the large tumor model. Further, we showed that HPV16 E6/E7 vaccines which contain previously described mutations that inhibit E6 and E7 oncogenic properties, or contain only predicted immunodominant epitopes, maintained the ability to control HPV16+ tumorigenesis through to the study end on day 49. This effect was especially pronounced in the rAd5-16/E6E7_Mt_ group, with complete regression of tumors in all mice and lasting regression in half of the mice. These findings support the further development of mucosally administered therapeutic adenovirus vaccines against HPV.

Antigen-specific cytotoxic CD8+ T cells play critical roles in immunotherapies [51] and thus it is important that any therapeutic vaccine be able to generate this type of response. We sought to understand how T cell dynamics changed in response to antigen-specific vaccination. When examining the TILs generated by rAd5-16/E6E7_Wt_, we observed an increase in CD4+, CD8+, and Treg cells. Treg cells are typically associated with suppression of the effector T cell response [50]. We found that although there was an increase in Tregs infiltrating the tumor compared to tumors from control animals, the ratio of Treg/CD8+ T cells suggested a more cytotoxic tumor microenvironment and aligned with that observed in human subjects with better clinical outcomes [43]. Further, in the small tumor model, administration of the rAd5 encoding E6 and E7 with mutations did not appear to alter the overall distribution of CD4+ and CD8+ T cells, but rAd5 encoding E6E7 genes were able to generate antigen-specific CD8+ T_EM_ cells in the periphery, with rAd5-16/E6E7_Mt_ generating a more pronounced response than rAd5-16/E6E7_epi_.

A few limitations of this study can be considered. First, in humans this vaccine platform is typically delivered to the ileum via enterically coated tablets; it is possible that oral delivery in mice would expose rAd5 to the low pH of the stomach, interfering with the vaccine’s potency [41]. However, previous studies with rAd5 vaccines show agreement in immunological readouts when comparing intranasal and oral delivery in hamsters [27,42,52]. Another limitation of this study is the use of a subcutaneous tumor model when, in humans, HPV induces mucosal tumors. While this proof-of-concept experiment shows promise for biological relevance, future studies may include modeling these vaccines in orthotopic tumor models to better predict clinical efficacy [53].

The generation of antigen-specific T cells in humans is important in the control of HPV tumorigenesis. It has been suggested that the exclusion of antigen-specific CD8+ T cells from the epithelium may play a key role in the progression of intraepithelial neoplasia [54]. Further, α4β7+ CD8+ T cells are better able to enter the cervical mucosa and are associated with cervical lesion clearance compared to those excluded from the epithelium [54]. Studies with an electroporated DNA vaccine candidate suggested that the ability to elicit potent antigen-specific T cell responses correlated with histopathological regression and a reduction in HPV viral DNA detection [20]. The mucosal rAd5 VAAST platform employed here has been used to generate mucosal homing T cells in human clinical trials investigating the platform’s prophylactic effects. In a phase II influenza clinical trial comparing the immunogenicity and efficacy of this mucosal rAd5 compared to a licensed injected vaccine, rAd5 was able to generate significantly more T cells expressing the mucosal homing marker α4β7+ [22]. The generation of antigen-specific T cells has also been demonstrated with the VAAST platform in a phase I COVID-19 clinical trial where rAd5 generated more cytotoxic T cell responses compared to the responses generated by an injected mRNA vaccination [55]. Although the therapeutic, rather than prophylactic, efficacy of the VAAST platform has not yet been tested in humans, the ability to generate mucosal homing cytotoxic CD8+ T cells combined with the preclinical results described here suggest that this platform may be able to generate a potent and effective T cell response that targets the provenance of HPV tumorigenesis.

Overall, we show the generation of antigen-specific CD8+ T cells induced by therapeutic vaccination via a mucosal route while also demonstrating a reduction in tumor volume and increased survival in a murine model.

## 5. Conclusions

These proof-of-concept results suggest that the rAd5 platform provides a promising technology in humans to treat HPV-derived cervical dysplasia. Currently, several therapeutic vaccines for HPV have completed placebo-controlled clinical trials and have largely been administered via injection or electroporation. A systematic review of different HPV16 and HPV18 E6 and E7 vaccines demonstrated a total overall proportion of regression from CIN2/3 to CIN1 at 0.54 compared a placebo group at 0.27 [56]. These numbers represent a meaningful reduction in the advancement of cervical cancer; yet, there remains room for improvement. Future research into the clinical use of mucosal rAd5 as a therapeutic could include investigation of optimal dose regimes, including investigations into the spacing between doses to allow for optimal contraction and expansion of antigen-specific T cells. Further, it is tantalizing to imagine that a non-invasive treatment, like the one described here, could be administered early in cervical cancer diagnosis, even at the point of infection identification, before CIN2-3 is reached.

While treatment options exist for CIN2-3, these treatments can be invasive or require frequent interventions. In low- and middle-income countries (LMICs), the introduction of preventative HPV vaccination campaigns began later with only 41% of LMICs introducing these vaccines by 2019 [57], leaving the burden of infection falling more heavily in regions where access to healthcare services and cold chain logistics is limited. A vaccine strategy, like the one described here, which can be therapeutically administered to activate necessary mucosal immune responses, using an easy-to-administer thermostable tablet, could have an even greater global impact on the treatment of pre-cancerous lesions, thereby preventing the development of malignant cervical cancer, particularly in regions where healthcare access is limited.

## Data Availability

Raw data are available upon request for the purposes of reproducing or adding to the analysis except in cases where data or information encroaches on the intellectual property of Vaxart, Inc. All data discussed in this article are available in the main or Supplementary Materials.

## Supplementary Materials

The following supporting information can be downloaded at: https://www.mdpi.com/article/10.3390/vaccines12090955/s1. Supplementary Figure S1: rAd5 vaccines expressing WT and non-oncogenic E6E7 induce specific T cells in J:DO mice. Spot forming units (SFU) measuring IFNγ in rAd5-vaccinated J:DO mice after stimulation with media, E6, or E7 peptide pools. Mean and SEM, two-way ANOVA. *n* = 3–8 mice/group. *p* = 0.01 to 0.05 (*), *p* = 0.001 to 0.01 (**). Supplementary Figure S2: Individual tumor volumes of C57BL/6 mice bearing subcutaneous TC-1 tumors. Individual tumor volumes, measured in mm3, of the individual mice from the (A) small tumor model with rAd5-E6E7WT, (B) large tumor model with rAd5-16/E6E7WT, (C) large tumor model with rAd5-16/E6E7WT for TIL analysis and (D) small tumor model with rAd5-16/E6E7Mu and rAd5-16/E6E7epi. Supplementary Figure S3: rAd5 expressing non-oncogenic E6E7 does not change overall distribution of CD4+ and CD8+ T cells. (A) CD3+, (B) CD8+, (C) CD4+, (D) effector memory CD8+, (E) central memory CD8+, or (F) naïve CD8+ T cells did not alter in relative percentage between vaccination groups. Small differences were not statically significant unless specified *n* = 10 mice/group, mean and SEM, two-way ANOVA. *p* = 0.001 to 0.01 (**).

## References

[1] De Sanjose S., Serrano B., Tous S., Alejo M., Lloveras B., Quiros B., Clavero O., Vidal A., Ferrandiz-Pulido C., Pavon M.A. (2018). Burden of Human Papillomavirus (HPV)-Related Cancers Attributable to HPVs 6/11/16/18/31/33/45/52 and 58. JNCI Cancer Spectr..

[2] Muhr L.S.A., Eklund C., Dillner J. (2018). Towards quality and order in human papillomavirus research. Virology.

[3] Hildesheim A., Gonzalez P., Kreimer A.R., Wacholder S., Schussler J., Rodriguez A.C., Porras C., Schiffman M., Sidawy M., Schiller J.T. (2016). Impact of human papillomavirus (HPV) 16 and 18 vaccination on prevalent infections and rates of cervical lesions after excisional treatment. Am. J. Obstet. Gynecol..

[4] Ho G.Y., Bierman R., Beardsley L., Chang C.J., Burk R.D. (1998). Natural history of cervicovaginal papillomavirus infection in young women. N. Engl. J. Med..

[5] Ljubojevic S., Skerlev M. (2014). HPV-associated diseases. Clin. Dermatol..

[6] Khallouf H., Grabowska A.K., Riemer A.B. (2014). Therapeutic Vaccine Strategies against Human Papillomavirus. Vaccines.

[7] Hoffman S.R., Le T., Lockhart A., Sanusi A., dal Santo L., Davis M., McKinney D.A., Brown M., Poole C., Willame C. (2017). Patterns of persistent HPV infection after treatment for cervical intraepithelial neoplasia (CIN): A systematic review. Int. J. Cancer.

[8] Stern P.L., van der Burg S.H., Hampson I.N., Broker T.R., Fiander A., Lacey C.J., Kitchener H.C., Einstein M.H. (2012). Therapy of human papillomavirus-related disease. Vaccine.

[9] Cheng S., Schmidt-Grimminger D.C., Murant T., Broker T.R., Chow L.T. (1995). Differentiation-dependent up-regulation of the human papillomavirus E7 gene reactivates cellular DNA replication in suprabasal differentiated keratinocytes. Genes Dev..

[10] Moody C.A., Laimins L.A. (2010). Human papillomavirus oncoproteins: Pathways to transformation. Nat. Rev. Cancer.

[11] Chabeda A., Yanez R.J.R., Lamprecht R., Meyers A.E., Rybicki E.P., Hitzeroth I.I. (2018). Therapeutic vaccines for high-risk HPV-associated diseases. Papillomavirus Res..

[12] Borysiewicz L.K., Fiander A., Nimako M., Man S., Wilkinson G.W., Westmoreland D., Evans A.S., Adams M., Stacey S.N., Boursnell M.E. (1996). A recombinant vaccinia virus encoding human papillomavirus types 16 and 18, E6 and E7 proteins as immunotherapy for cervical cancer. Lancet.

[13] Ding Z., Ou R., Ni B., Tang J., Xu Y. (2013). Cytolytic activity of the human papillomavirus type 16 E711-20 epitope-specific cytotoxic T lymphocyte is enhanced by heat shock protein 110 in HLA-A*0201 transgenic mice. Clin. Vaccine Immunol..

[14] Juarez V., Pasolli H.A., Hellwig A., Garbi N., Arregui A.C. (2012). Virus-Like Particles Harboring CCL19, IL-2 and HPV16 E7 Elicit Protective T Cell Responses in HLA-A2 Transgenic Mice. Open Virol. J..

[15] Khan S., Oosterhuis K., Wunderlich K., Bunnik E.M., Bhaggoe M., Boedhoe S., Karia S., Steenbergen R.D.M., Bosch L., Serroyen J. (2017). Development of a replication-deficient adenoviral vector-based vaccine candidate for the interception of HPV16- and HPV18-induced infections and disease. Int. J. Cancer.

[16] Lee S.J., Yang A., Wu T.C., Hung C.F. (2016). Immunotherapy for human papillomavirus-associated disease and cervical cancer: Review of clinical and translational research. J. Gynecol. Oncol..

[17] McCarthy C., Youde S.J., Man S. (2006). Definition of an HPV18/45 cross-reactive human T-cell epitope after DNA immunisation of HLA-A2/KB transgenic mice. Int. J. Cancer.

[18] Perez S., Zimet G.D., Tatar O., Stupiansky N.W., Fisher W.A., Rosberger Z. (2018). Human Papillomavirus Vaccines: Successes and Future Challenges. Drugs.

[19] Rice A.E., Latchman Y.E., Balint J.P., Lee J.H., Gabitzsch E.S., Jones F.R. (2015). An HPV-E6/E7 immunotherapy plus PD-1 checkpoint inhibition results in tumor regression and reduction in PD-L1 expression. Cancer Gene Ther..

[20] Trimble C.L., Trimble C.L., Morrow M.P., Kraynyak K.A., Shen X., Dallas M., Yan J., Edwards L., Parker R.L., Denny L. (2015). Safety, efficacy, and immunogenicity of VGX-3100, a therapeutic synthetic DNA vaccine targeting human papillomavirus 16 and 18 E6 and E7 proteins for cervical intraepithelial neoplasia 2/3: A randomised, double-blind, placebo-controlled phase 2b trial. Lancet.

[21] Wieking B.G., Vermeer D.W., Spanos W.C., Lee K.M., Vermeer P., Lee W.T., Xu Y., Gabitzsch E.S., Balcaitis S., Balint J.P. (2012). A non-oncogenic HPV 16 E6/E7 vaccine enhances treatment of HPV expressing tumors. Cancer Gene Ther..

[22] McIlwain D.R., Chen H., Rahil Z., Bidoki N.H., Jiang S., Bjornson Z., Kolhatkar N.S., Martinez C.J., Gaudilliere B., Hedou J. (2021). Human influenza virus challenge identifies cellular correlates of protection for oral vaccination. Cell Host Microbe.

[23] Liebowitz D., Gottlieb K., Kolhatkar N.S., Garg S.J., Asher J.M., Nazareno J., Kim K., McIlwain D.R., Tucker S.N. (2020). Efficacy, immunogenicity, and safety of an oral influenza vaccine: A placebo-controlled and active-controlled phase 2 human challenge study. Lancet Infect. Dis..

[24] Kim L., Liebowitz D., Lin K., Kasparek K., Pasetti M.F., Garg S.J., Gottlieb K., Trager G., Tucker S.N. (2018). Safety and immunogenicity of an oral tablet norovirus vaccine, a phase I randomized, placebo-controlled trial. JCI Insight.

[25] Kim L., Martinez C.J., Hodgson K.A., Trager G.R., Brandl J.R., Sandefer E.P., Doll W.J., Liebowitz D., Tucker S.N. (2016). Systemic and mucosal immune responses following oral adenoviral delivery of influenza vaccine to the human intestine by radio controlled capsule. Sci. Rep..

[26] Liebowitz D., Lindbloom J.D., Brandl J.R., Garg S.J., Tucker S.N. (2015). High titre neutralising antibodies to influenza after oral tablet immunisation: A phase 1, randomised, placebo-controlled trial. Lancet Infect. Dis..

[27] Johnson S., Martinez C.I., Tedjakusuma S.N., Peinovich N., Dora E.G., Birch S.M., Kajon A.E., Werts A.D., Tucker S.N. (2022). Oral Vaccination Protects Against Severe Acute Respiratory Syndrome Coronavirus 2 in a Syrian Hamster Challenge Model. J. Infect. Dis..

[28] Flitter B.A., Braun M.R., Tucker S.N. (2022). Drop the Needle; A Temperature Stable Oral Tablet Vaccine Is Protective against Respiratory Viral Pathogens. Vaccines.

[29] Cummings J.F., Tucker S. Potent Immune Responses to Norovirus G1.1 Evaluated in Elderly Subjects following Oral Tablet Delivery in a Phase 1 Placebo-Controlled Study. Proceedings of the World Vaccine Congress 2022.

[30] Johnson S., Martinez C.I., Jegede C.B., Gutierrez S., Cortese M.C., Martinez J., Garg S.J., Peinovich N., Dora E.G., Tucker S.N. (2022). SARS-CoV-2 oral tablet vaccination induces neutralizing mucosal IgA in a phase 1 open label trial. medRxiv.

[31] Ribas A., Wolchok J.D. (2018). Cancer immunotherapy using checkpoint blockade. Science.

[32] Li C., Cang W., Gu Y., Chen L., Xiang Y. (2023). The anti-PD-1 era of cervical cancer: Achievement, opportunity, and challenge. Front. Immunol..

[33] Callahan M.K., Postow M.A., Wolchok J.D. (2014). CTLA-4 and PD-1 Pathway Blockade: Combinations in the Clinic. Front. Oncol..

[34] Chen Z., Pang N., Du R., Zhu Y., Fan L., Cai D., Ding Y., Ding J. (2016). Elevated Expression of Programmed Death-1 and Programmed Death Ligand-1 Negatively Regulates Immune Response against Cervical Cancer Cells. Mediat. Inflamm..

[35] Fu J., Malm I.J., Kadayakkara D.K., Levitsky H., Pardoll D., Kim Y.J. (2014). Preclinical evidence that PD1 blockade cooperates with cancer vaccine TEGVAX to elicit regression of established tumors. Cancer Res..

[36] Peng S., Tan M., Li Y.D., Cheng M.A., Farmer E., Ferrall L., Gaillard S., Roden R.B.S., Hung C.F., Wu T.C. (2021). PD-1 blockade synergizes with intratumoral vaccination of a therapeutic HPV protein vaccine and elicits regression of tumor in a preclinical model. Cancer Immunol. Immunother..

[37] Mkrtichyan M., Chong N., Abu Eid R., Wallecha A., Singh R., Rothman J., Khleif S.N. (2013). Anti-PD-1 antibody significantly increases therapeutic efficacy of Listeria monocytogenes (Lm)-LLO immunotherapy. J. Immunother. Cancer.

[38] Roberts J.N., Buck C.B., Thompson C.D., Kines R., Bernardo M., Choyke P.L., Lowy D.R., Schiller J.T. (2007). Genital transmission of HPV in a mouse model is potentiated by nonoxynol-9 and inhibited by carrageenan. Nat. Med..

[39] Berraondo P., Nouze C., Preville X., Ladant D., Leclerc C. (2007). Eradication of large tumors in mice by a tritherapy targeting the innate, adaptive, and regulatory components of the immune system. Cancer Res..

[40] Lin K.Y., Guarnieri F.G., Staveley-O’Carroll K.F., Levitsky H.I., August J.T., Pardoll D.M., Wu T.C. (1996). Treatment of established tumors with a novel vaccine that enhances major histocompatibility class II presentation of tumor antigen. Cancer Res..

[41] McConnell E.L., Basit A.W., Murdan S. (2008). Measurements of rat and mouse gastrointestinal pH, fluid and lymphoid tissue, and implications for in-vivo experiments. J. Pharm. Pharmacol..

[42] Braun M.R., Martinez C.I., Dora E.G., Showalter L.J., Mercedes A.R., Tucker S.N. (2023). Mucosal immunization with Ad5-based vaccines protects Syrian hamsters from challenge with omicron and delta variants of SARS-CoV-2. Front. Immunol..

[43] Jordanova E.S., Gorter A., Ayachi O., Prins F., Durrant L.G., Kenter G.G., van der Burg S.H., Fleuren G.J. (2008). Human leukocyte antigen class I, MHC class I chain-related molecule A, and CD8+/regulatory T-cell ratio: Which variable determines survival of cervical cancer patients?. Clin. Cancer Res..

[44] Nielsen M., Lundegaard C., Worning P., Lauemoller S.L., Lamberth K., Buus S., Brunak S., Lund O. (2003). Reliable prediction of T-cell epitopes using neural networks with novel sequence representations. Protein Sci..

[45] Lundegaard C., Lamberth K., Harndahl M., Buus S., Lund O., Nielsen M. (2008). NetMHC-3.0: Accurate web accessible predictions of human, mouse and monkey MHC class I affinities for peptides of length 8-11. Nucleic Acids Res..

[46] Scallan C.D., Tingley D.W., Lindbloom J.D., Toomey J.S., Tucker S.N. (2013). An adenovirus-based vaccine with a double-stranded RNA adjuvant protects mice and ferrets against H5N1 avian influenza in oral delivery models. Clin. Vaccine Immunol..

[47] Simpson-Herren L., Lloyd H.H. (1970). Kinetic parameters and growth curves for experimental tumor systems. Cancer Chemother. Rep..

[48] Boursnell M.E., Rutherford E., Hickling J.K., Rollinson E.A., Munro A.J., Rolley N., McLean C.S., Borysiewicz L.K., Vousden K., Inglis S.C. (1996). Construction and characterisation of a recombinant vaccinia virus expressing human papillomavirus proteins for immunotherapy of cervical cancer. Vaccine.

[49] IEDB Analysis Resource. http://tools.iedb.org/main/tcell/.

[50] Scott E.N., Gocher A.M., Workman C.J., Vignali D.A.A. (2021). Regulatory T Cells: Barriers of Immune Infiltration into the Tumor Microenvironment. Front. Immunol..

[51] Raskov H., Orhan A., Christensen J.P., Gogenur I. (2021). Cytotoxic CD8(+) T cells in cancer and cancer immunotherapy. Br. J. Cancer.

[52] Langel S.N., Johnson S., Martinez C.I., Tedjakusuma S.N., Peinovich N., Dora E.G., Kuehl P.J., Irshad H., Barrett E.G., Werts A.D. (2022). Adenovirus type 5 SARS-CoV-2 vaccines delivered orally or intranasally reduced disease severity and transmission in a hamster model. Sci. Transl. Med..

[53] Zottnick S., Voss A.L., Riemer A.B. (2020). Inducing Immunity Where It Matters: Orthotopic HPV Tumor Models and Therapeutic Vaccinations. Front. Immunol..

[54] Trimble C.L., Clark R.A., Thoburn C., Hanson N.C., Tassello J., Frosina D., Kos F., Teague J., Jiang Y., Barat N.C. (2010). Human papillomavirus 16-associated cervical intraepithelial neoplasia in humans excludes CD8 T cells from dysplastic epithelium. J. Immunol..

[55] Tucker S.N. Oral Tablet Vaccination to SARS-CoV-2 Induces Long Lasting Cross-reactive Mucosal Antibody Responses in Humans. Proceedings of the World Vaccine Congress 2022.

[56] Ibrahim Khalil A., Zhang L., Muwonge R., Sauvaget C., Basu P. (2023). Efficacy and safety of therapeutic HPV vaccines to treat CIN 2/CIN 3 lesions: A systematic review and meta-analysis of phase II/III clinical trials. BMJ Open.

[57] Bruni L., Saura-Lazaro A., Montoliu A., Brotons M., Alemany L., Diallo M.S., Afsar O.Z., LaMontagne D.S., Mosina L., Contreras M. (2021). HPV vaccination introduction worldwide and WHO and UNICEF estimates of national HPV immunization coverage 2010–2019. Prev. Med..

