# Supplementary material for: Elimination of Human Papillomavirus 16-Positive Tumors by a Mucosal rAd5 Therapeutic Vaccination in a Pre-Clinical Murine Study
Source: Vaccines (Basel). 2024 Aug 23;12(9):955. doi: 10.3390/vaccines12090955 (PMC11435741; doi:10.3390/vaccines12090955)
Supplement: Supplementary file 1 [file vaccines-12-00955-s001.zip › vaccines-3110661-supplementary.pdf]

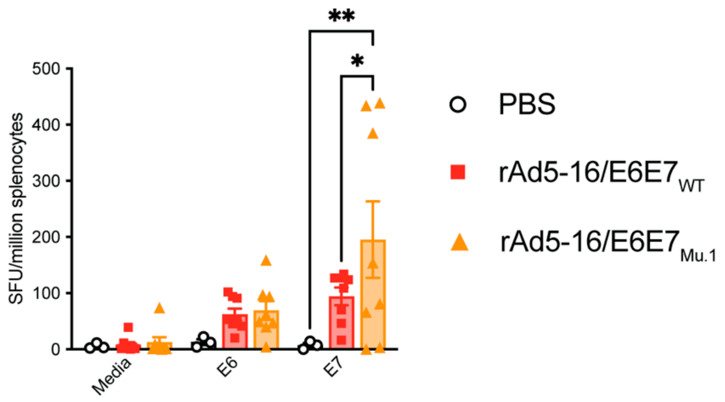

Supplementary Figure S1: rAd5 vaccines expressing WT and non-oncogenic E6E7 induce specific T cells in J:DO mice. Spot forming units (SFU) measuring IFNγ in rAd5-vaccinated J:DO mice after stimulation with media, E6, or E7 peptide pools. Mean and SEM, two-way ANOVA. n=3-8 mice/group. p = 0.01 to 0.05 (*), p = 0.001 to 0.01 (**)

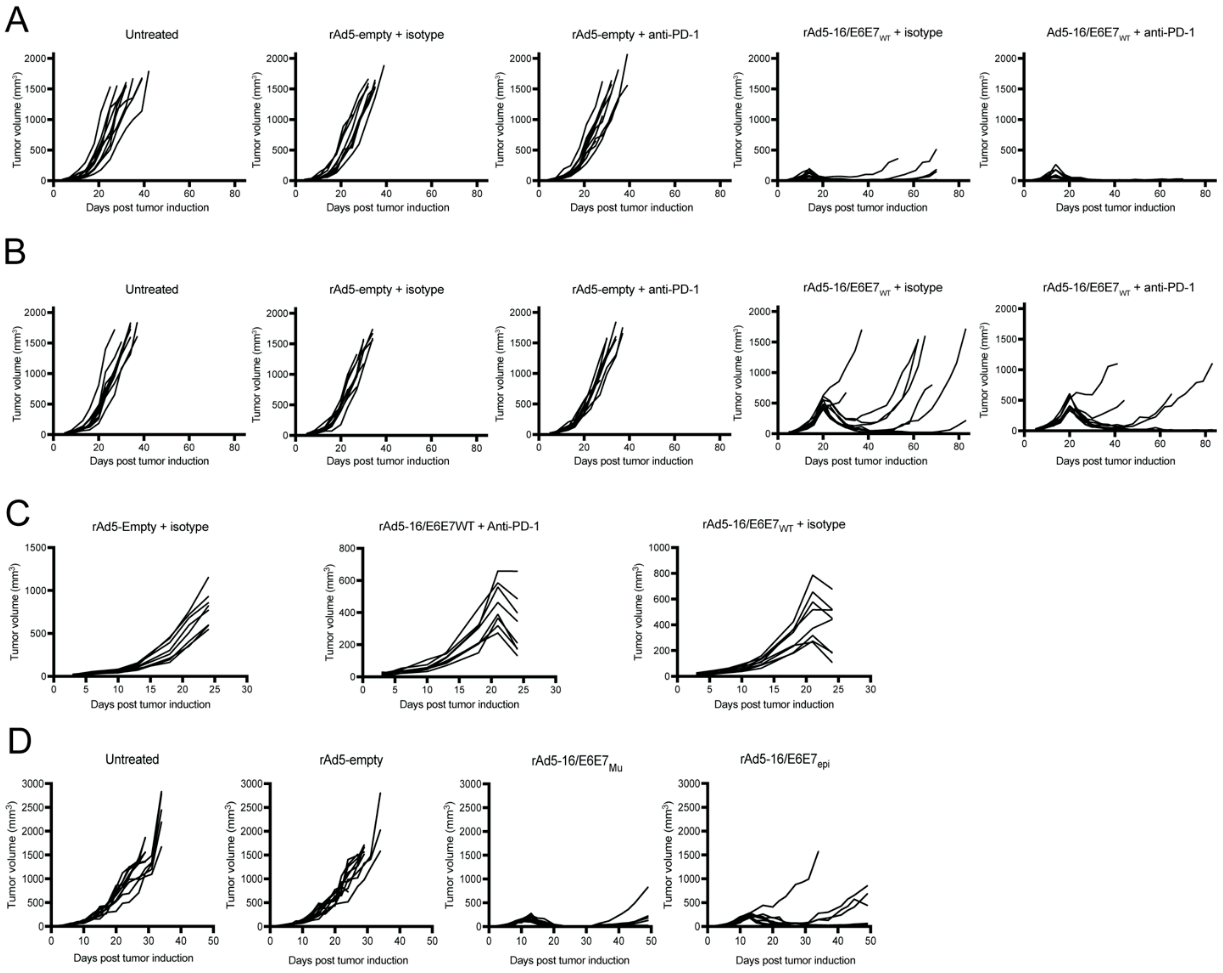

Supplementary Figure S2: Individual tumor volumes of C57BL/6 mice bearing subcutaneous TC-1 tumors. Individual tumor volumes, measured in mm^3, of the individual mice from the (A) small tumor model with rAd5-E6E7$_{WT}$, (B) large tumor model with rAd5-16/E6E7$_{WT}$, (C) large tumor model with rAd5-16/E6E7$_{WT}$ for TIL analysis and (D) small tumor model with rAd5-16/E6E7$_{Mu}$ and rAd5-16/E6E7$_{epi}$.

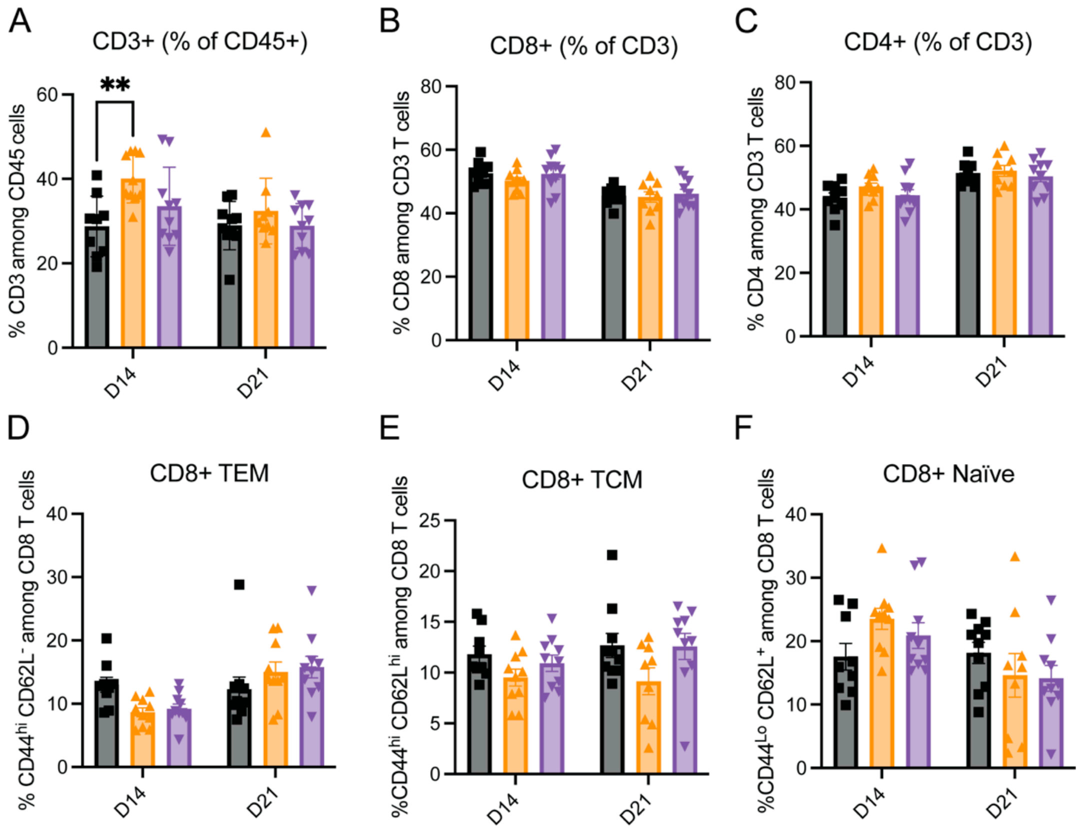

Supplementary Figure S3: rAd5 expressing non-oncogenic E6E7 does not change overall distribution of CD4+ and CD8+ T cells. (A) CD3+, (B) CD8+, (C) CD4+, (D) effector memory CD8+, (E) central memory CD8+, or (F) naïve CD8+ T cells did not alter in relative percentage between vaccination groups. Small differences were not statically significant unless specified n=10 mice/group, mean and SEM, two-way ANOVA. p = 0.001 to 0.01 (**)
